# Characterization and classification of ductal carcinoma tissue using four channel based stokes-mueller polarimetry and machine learning

**DOI:** 10.1007/s10103-024-04056-5

**Published:** 2024-05-04

**Authors:** Spandana KU, Sindhoora Kaniyala Melanthota, Raghavendra U, Sharada Rai, K. K. Mahato, Nirmal Mazumder

**Affiliations:** 1 Department of Biophysics, Manipal School of Life Sciences, Manipal Academy of Higher Education, Manipal, 576104 India; 2 Department of Instrumentation and Control Engineering, Manipal Institute of Technology, Manipal Academy of Higher Education, Manipal, Karnataka 576104 India; 3 Department of Pathology, Kasturba Medical College, Mangalore, Karnataka 575001 India

**Keywords:** Stokes vector, Mueller matrix, Polarization, Tissue, Polar decomposition, Machine learning

## Abstract

Interaction of polarized light with healthy and abnormal regions of tissue reveals structural information associated with its pathological condition. Even a slight variation in structural alignment can induce a change in polarization property, which can play a crucial role in the early detection of abnormal tissue morphology. We propose a transmission-based Stokes-Mueller microscope for quantitative analysis of the microstructural properties of the tissue specimen. The Stokes-Mueller based polarization microscopy provides significant structural information of tissue through various polarization parameters such as degree of polarization (DOP), degree of linear polarization (DOLP), and degree of circular polarization (DOCP), anisotropy (r) and Mueller decomposition parameters such as diattenuation, retardance and depolarization. Further, by applying a suitable image processing technique such as Machine learning (ML) output images were analysed effectively. The support vector machine image classification model achieved 95.78% validation accuracy and 94.81% testing accuracy with polarization parameter dataset. The study's findings demonstrate the potential of Stokes-Mueller polarimetry in tissue characterization and diagnosis, providing a valuable tool for biomedical applications.

**Supplementary Information:**

The online version contains supplementary material available at 10.1007/s10103-024-04056-5.

## Introduction

 Non-invasive optical techniques play a pivotal role in modern biomedical diagnostics, necessitating a comprehensive grasp of light-tissue interactions and the scattering phenomena from microstructures, crucial for discerning both normal and pathological regions [1–3]. Optical properties of biological tissue such as refractive index, imparting scattering phenomenon could reveal valuable information for optical approaches such as early stage and pre-cancer diagnosis [4]. Unfortunately, the variations in light tissue interaction for normal and abnormal tissues are not clearly understood by available conventional techniques [5]. For example, several biological structures are birefringent in nature, thus by determining various aspects of polarization property, one can gain knowledge about the molecular nature of the sample [6, 7]. Polarized light microscopy with an ability for selective visualization of anisotropic structures, has been a great help for pathologists. It provides real-time and label-free imaging of biological structures [8]. Optical polarization techniques are widely incorporated in spectroscopy/microscopy with various light sources such as mercury lamps, light emitting diodes (LED) and lasers. The technique was widely used in differentiating active and affected tuberculous focus, for hyaline change studies and several other optical fundoscopic examinations [9].

Researchers around the world are working on several aspects of polarization-based imaging techniques [10] and remain to be a potential diagnostic tool in the field of biomedicine for abnormal tissue detection in the liver, skin, oesophagus, bladder, cervix, colon and so on [11, 12]. The polarization state of light was explicitly described using Stokes vector, whereas, polarization property of the sample responsible for the change in polarization state of light from input Stokes vector (S_in_) to output Stokes vector (S_out_) was described using Mueller matrix (M) [13]. Several linear optical measurements have widely employed the Stokes- Mueller formalism. However, the occurrence of several scattering effects in complex mediums such as biological tissues leads to complexities in the measurement. In this regard, the assessment of biological tissue using polar decomposition approach, where the decomposition of Mueller matrix into three basis matrices was used as an effective tool to distinguish multiple scattered lights and to gain individual polarization properties of the sample. Polarization properties such as retardance, diattenuation and depolarization can be used for investigating the composition and microstructure of biological tissue, which could be beneficial in image-guided therapy and tissue diagnosis [14]. Mueller matrix imaging significantly enhances fibrous structures when compared to conventional polarization imaging. The technique being non-invasive, gaining greater importance as a diagnostic tool since it can provide several polarization properties when compared to conventional microscopy [15].

Polarization imaging, mainly Mueller polarimetry, has various unique advantages as in situ and non-contact techniques for identifying tissue microstructures [1, 16–19]. In a study proposed by Arteaga et al., the polarization property of the specimen was completely extracted by Mueller matrix microscopy based on two continuously rotating wave plates [11]. Furthermore, comprehensive characterization of the polarization properties of the sample using the Mueller matrix; is found to have increasing application in textiles [20], in the characterization of biological tissues [21], plasmonic nanoparticles and other various structures. Biomedical studies of several pathological tissues, such as cervical cancer [15, 17], colon cancer [22], liver fibrosis [23] and skin cancer [24] have shown the potential of the Mueller matrix parameters in diagnosis. Mazumder et al., have developed a four-channel photon counting polarization microscopy, based on Stokes polarimetry to determine the complete polarization states of second harmonic (SH) lights from the anisotropic samples [25, 26]. It was noticed that collagen-rich structures show high contrast with polarized light due to the birefringence property of the collagen fibers. Hence, the change in tissue structures, particularly the collagen contents and organization can be correlated with tissue pathology using the Stokes polarimetry module. Further, collagen production in cancer tissue could be identified and inspected by determining the degree of linear polarization (DOLP), degree of circular polarization (DOCP), and angle of polarization (AOP) [25, 26]. However, the SH signal being very specific to non-centrosymmetric molecules, in particular, collagen in pathological tissue samples, limits the use of the technique for biomedical applications [27]. Hence, we propose a four-channel linear Stokes-Mueller polarimetric module to study tissue properties irrespective of its non-centrosymmetric nature [19].

Breast cancer, the most common cancer found in women [28], among 50–75% of patients, it occurs in the cell lining of the duct walls and is referred to as ductal carcinoma. Based on the appearance of tumour cells under the microscope, pathologist grade cancers, and suggests treatment options and estimated outcomes. Several medical imaging modalities are involved in the screening, diagnosis, treatment planning and monitoring of breast cancer. However, various studies have shown the importance of the structural morphology of the tumour since it possesses clinically significant information [29]. Histopathology has been the gold standard technique for cancer diagnosis and stroma was found to play a major role in the behaviour and response of cancer to the therapy, yet the methods available for stromal architecture assessment are often qualitative and subjective [30, 31]. Hence, to perform quantitative polarimetry, this study proposes a cost-effective, transmission-based four channel Stokes-Mueller microscope with LED as a light source for analysis of microstructural properties of the breast tissue sample.

## Materials and methods

### Theoretical background

When polarized light is allowed to pass through micro-meter depth of the sample, the polarization state of the incoming light is distorted. Polarization analysis can be carried out by Jones calculus as well as Stokes algebra. Again, Jones calculus is limited to fully polarized light whereas Stokes algebra is applied to all polarization states (partially polarized, unpolarized and fully polarized). The polarization state of light can be described using the Stokes vector as follows,

1 $$S=\left[\begin{array}{c}{S}_{0}\\ {S}_{1}\\ {S}_{2}\\ {S}_{3}\end{array}\right]=\left[\begin{array}{c}{I}_{0}+{I}_{90}\\ {I}_{0}-{I}_{90}\\ {I}_{45}-{I}_{135}\\ {I}_{RCP}-{I}_{LCP}\end{array}\right]$$

Where, first parameter S_0_ describes the total optical field intensity, S_1_ describes the difference in intensity between 0^0^ and 90^0^ linearly polarized states, the intensity difference between 45^0^ and -45^0^ linearly polarized states are described by S_2_ and the difference in intensity between the right and left-handed circularly polarized states are described by S_3_. DOP, DOLP, DOCP and anisotropy (r) of light are defined by the following equations:

2 $$\text{DOP}=\frac{\sqrt{\left({\text{S}}_1^2+{\text{S}}_2^2+{\text{S}}_3^2\right)}}{{\text{S}}_0};\;\text{DOLP}=\frac{\sqrt{\left({\text{S}}_1^2+{\text{S}}_2^2\right)}}{{\text{S}}_0};\;\mathrm{DOCP}=\frac{{\text{S}}_3}{{\text{S}}_0};\;\mathrm r=\frac{{2S}_1}{{3S}_0-S_1}$$

DOP represents the polarization property of the light, whose value ranges from 0 to 1. For perfectly polarized light, DOP = 1 and for unpolarized light, Stokes parameter, S_0_ = 1, S_1_ = S_2_ = S_3_ = 0 and DOP = 0. Depending on the degree of polarization, the DOP value ranges between 0 and 1 for partially polarized light. DOLP represents the crystalline alignment of molecules parallel to the linear polarization states and the value ranges between 0 and 1. Within the focal volume, the ability of molecules to flip the circularly scattered light is represented by DOCP and the value ranges between 0 and 1. The signal anisotropy is represented by ‘r’ and the value ranges between − 0.5 to 1. The Optical property of the sample responsible for the change in the polarization state of light after its interaction with an optical system can be described by the Mueller matrix [32, 33]. Input Stokes vector ($${S}_{in})$$ and output Stokes vector ($${S}_{out})$$ of light can be related as follows,

3 $$S_{out}=\text{M}.\;S_{in}$$

Then, sample Mueller matrix (M) can be measured as,

4 $${\text{M}}={S}_{out}{\cdot {S}_{in}}^{-1}$$

However, the cryptic nature of 16 element Mueller matrix, leaves behind an unclear understanding of polarization interactions [34]. Hence, it's necessary to decompose the Mueller matrix to gain insight into various properties such as diattenuation, retardance and depolarization. For turbid media such as biological tissue, ‘M’ can be decomposed using Lu-Chipman polar decomposition [35] into three basis matrices as

5 $$M={ M}_{\Delta }{ M}_{R}{M}_{D}$$

Where ${M}_{\Delta }$ is depolarization matrix, ${M}_{R}$ is retardance matrix and ${M}_{D}$ is diattenuation matrix, respectively.

### Stokes Mueller polarimetry

**Fig. 1:**
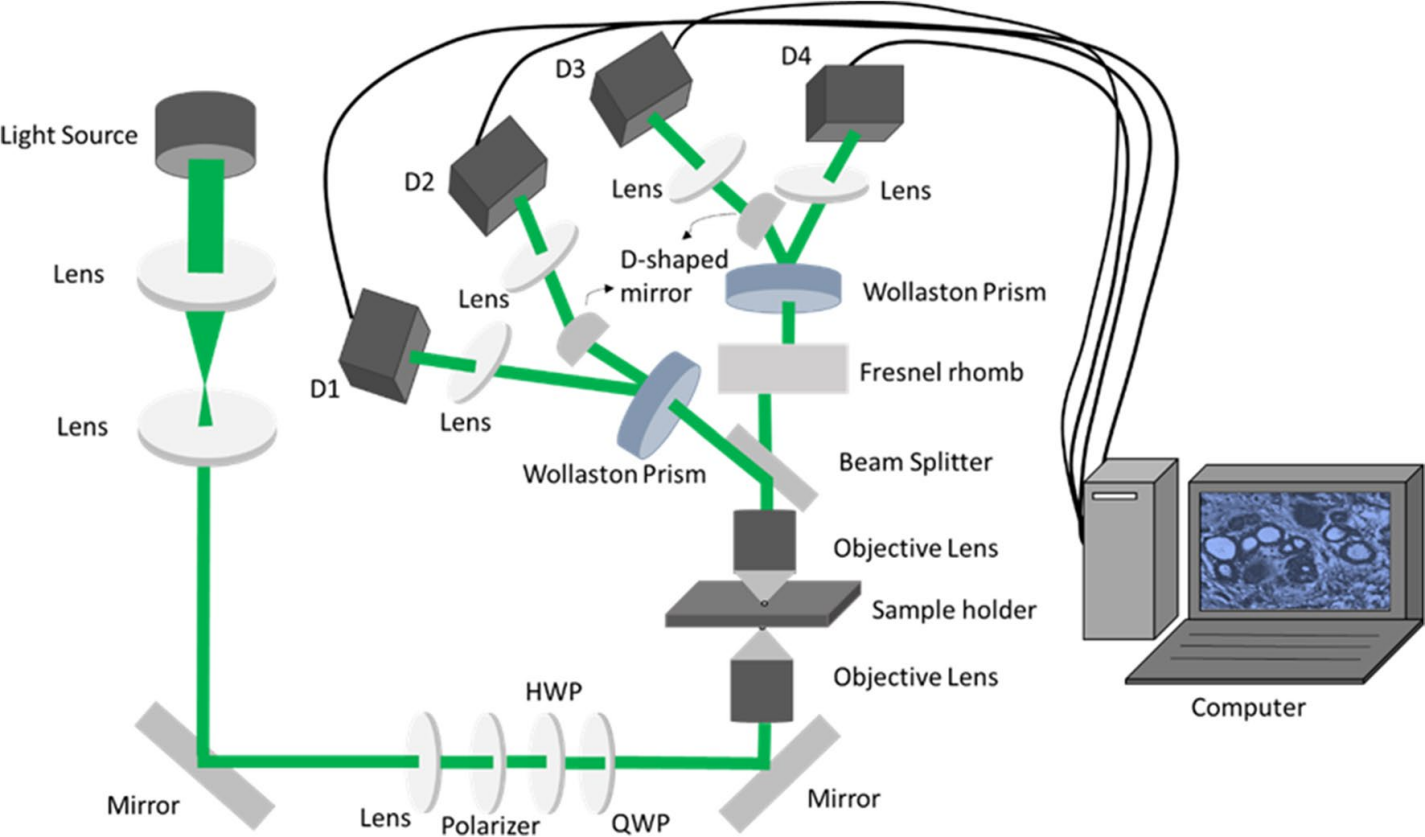
Polarization-resolved four-channel Stokes-Mueller polarimeter setup: HWP: half wave-plate, QWP: quarter wave-plate, D1, D2, D3, D4: CMOS camera

The polarization-sensitive imaging system is developed which consists of a LED as the light source (SOLIS-525C, Thorlabs, USA) controlled by a driver (DC2200, Thorlabs, USA), followed by a polarization state generator (PSG), polarization state analyzer (PSA) and detection system [36]. Light from the LED operating at 525 nm was set to have desired polarization state using PSG, consisting of a lens (AC254-040-A-ML, Thorlabs, USA), polarizer (LPVISA050-MP2, Thorlabs, USA), half wave plate (AHWP05M-600, Thorlabs, USA) and quarter wave plate (AQWP05M-600). The polarized light generated by PSG is passed through an objective lens (20X magnification, N1492800, Olympus, Japan) and then focused on to sample, vertically mounted on the sample holder. Transmitted light after the sample enters PSA and passed through the second objective lens (40X magnification N1479800, Olympus, Japan). Polarization state analyzer comprises a beam splitter, Fresnel’s rhomb (FR600QM, Thorlabs, USA), Wollaston prism (WP10, Thorlabs, USA), lenses and a D-shaped mirror (BBD1-E02, Thorlabs, USA). The beam splitter splits the beam into transmitted and reflected arms. Both the beams were allowed to pass through the combination of Wollaston prism, Fresnel’s rhomb, and lens system before reaching four complementary metal-oxide semiconductor (CMOS) cameras (DCC3260M, Thorlabs, USA), where Wollaston prism splits the beam into two with 20^0^ separation angle. The principle behind the technique remains in probing the change of polarization state and simultaneous measurement of four transmitted beams. A schematic representation of the polarization-based microscopic setup is shown in Fig. 1.

The optical components such as polarizers, half wave plate, quarter wave plate, Wollaston prism and Fresnel rhomb were calibrated to find their minimum and maximum intensity positions. Further, desired input polarization state was set at PSG and the output Stokes vector was measured. For 0^0^ polarization state, the input Stokes vector is [1 1 0 0]^T^ and for air as a sample, the output polarization state must be [1 1 0 0]^T^, since air does not change the polarization state of light. However, the variation in output polarization state values corresponds to the instrumental error. The Stokes-Mueller polarimeter was optimized by measuring the instrument matrix which should have a low condition number.

### Sample preparation

The human breast cancer samples were obtained from Kasturba Medical Hospital (KMC), Mangalore, India, after prior approval from Institutional Animal Ethics Committee (IAEC), KMC, Mangalore, India. The samples, procured after breast cancer surgery, were histologically identified as invasive ductal carcinoma. The obtained tissue samples were used for block preparation by cutting into pieces (thickness ~ 5 µm), fixed in formalin, dehydrated, and embedded in paraffin wax. Before imaging, the slices were unfrozen and sandwiched between two microscope cover slides together with some phosphate buffer saline (PBS) droplets to maintain the natural tissue osmolarity. Tissue samples comprise both tumour and healthy tissues. Once the slides are imaged with a developed polarization setup, the same slides were stained with hematoxylin and eosin (H&E) and the images were captured using a bright-field optical microscope.

### Development of image processing algorithm

The images acquired by the CMOS cameras were analyzed pixel by pixel to calculate individual intensity patterns that correspond to the Stokes vector, 16 components of the Mueller matrix as well as various polarization parameters. These parameters form the input dataset and were used to train the support vector machine (SVM) which is a supervised machine learning (ML) classification model [37–39]. In the present study, both normal and tumour regions of ductal carcinoma tissue were imaged under the Stokes-Mueller polarization setup with various input polarization states. Further, the polarization parameters such as DOP, DOLP, DOCP, anisotropy and Mueller matrix polar decomposition (MMPD) parameters were reconstructed using the MATLAB platform. Data augmentation was performed to increase the dataset size by flipping the images in horizontal and vertical directions, further each image is segmented into 16 equal parts. From each of these image patches, a gray level co-occurrence matrix (GLCM) is constructed which is an 8 × 8 matrix, that provides information regarding the co-occurrence of pixel values in the given image. 20 GLCM parameters such as contrast, correlation, energy, entropy etc. [40, 41] of normal and tumour regions of tissue are calculated. Each polarization parameter was considered individually while training the classifier to identify the significance of the specific polarization state of light in cancer detection.

## Results and discussion

### Stokes vector analysis

**Fig. 2:**
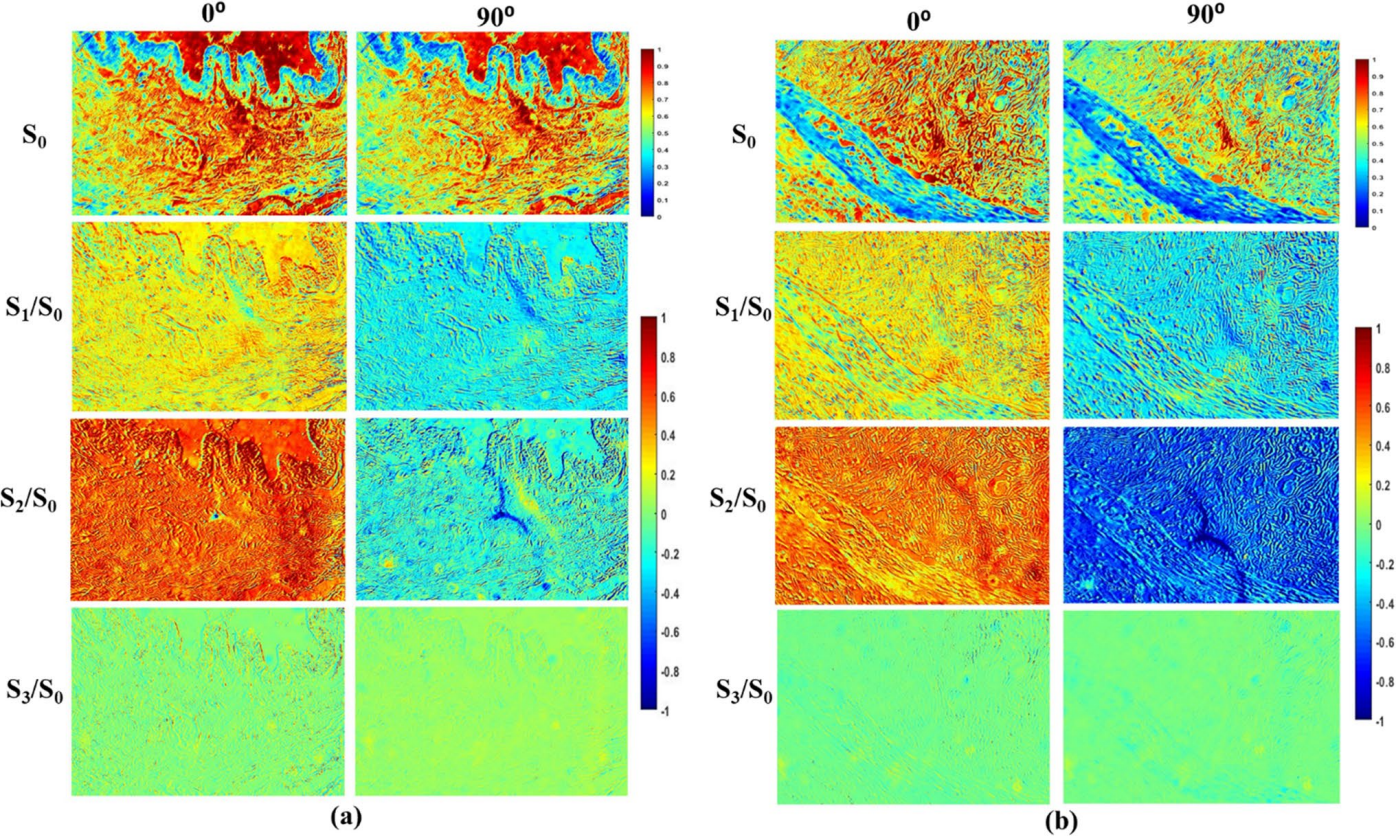
Reconstructed Stokes vector 2D images from (**a**) normal regions of ductal carcinoma samples, and (**b**) tumour regions of ductal carcinoma samples with the input polarization of 0^0^ and 90^0^. The color bar has a value ranging from 0 to 1 for S_0_ and -1 to 1 for the remaining parameters

The normal and tumour regions of ductal carcinoma tissue samples are used in this study. For a comparison, normal and tumour regions of breast ductal carcinoma tissue images were captured using both brightfield (Olympus BX51) and the developed polarization microscope, as shown in Supplementary Figure S1. The image captured using the developed polarization microscope shows better contrast when compared to the image captured using a bright field microscope. The breast ductal carcinoma tissue samples were illuminated with light of 0^0^, 90^0^, 45^0^, and right circular polarization (RCP) states and the output Stokes vector was measured, respectively. For 0^0^ input polarization state, input Stokes vector, S_in_ = [1, 1, 0, 0]^T^. However, after interaction with the sample, the output Stokes vector measured was S_out_ = [1, 0.8974, -0.3430, 0.0110]^T^. The difference in output Stokes vector is due to the change in the state of polarization after light-tissue interaction. 2D Stokes vector images were reconstructed for output states using MATLAB. Similarly, the process was repeated for other input polarization states such as 90^0^, 45^0^ and RCP. The output Stokes vector images (S_0_, S_1_, S_2_ and S_3_) for 0^0^ and 90^0^ input polarization are shown in Fig. 2.

**Fig. 3:**
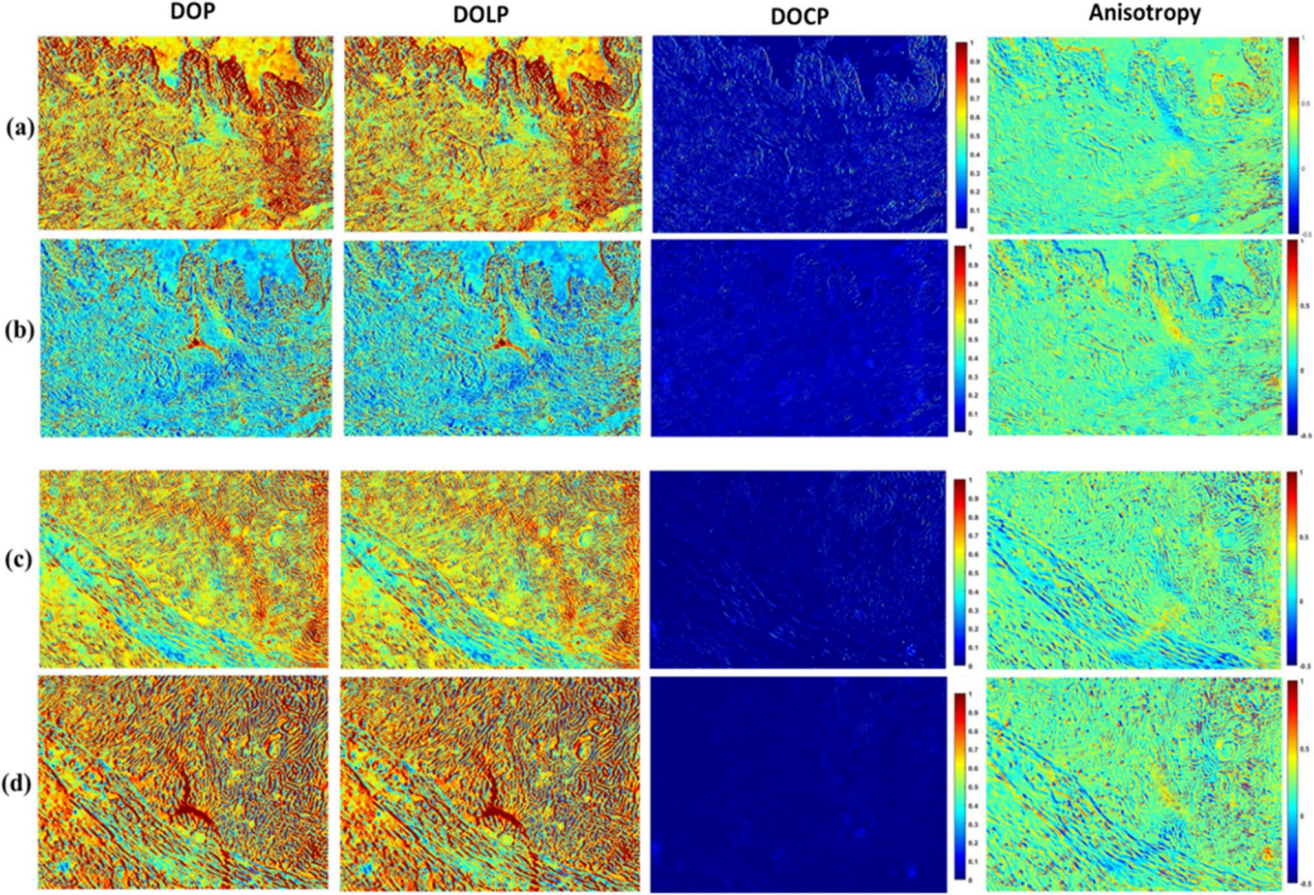
Polarization parameter images such as DOP, DOLP, DOCP and anisotropy. (**a**, **b**) For the normal regions and (**c**, **d**) for tumour regions ductal carcinoma sample, incident with the light of 0^0^ and 90^0^ polarization state respectively. The colour bar for DOP, DOLP, and DOCP has a value ranging from 0 to 1. Anisotropy has a colour bar ranging from -0.5 to 1

The Stokes parameter, S_0_ exhibits positive values ranging from 0 to 1 for both normal and tumour regions of the tissue. The other Stokes parameters exhibit values ranging from -1 to 1, with blue indicating a negative value and red representing a positive value. The Stokes images represent the dependence of polarization signal on the tissue architecture mainly due to the collagen fibre orientation. For both 0^0^ and 90^0^ polarization, the S_2_/S_0_ images for the normal region exhibit greater values than that of tumour regions. From Fig. 2, it is evident that the S_3_/S_0_ parameter for both normal and tumour breast tissue is nearly equivalent to zero when the sample is incident with 0^0^ and 90^0^ linearly polarized light. In addition, the reduced S_3_/S_0_ value for the tumour region compared to normal regions indicates the lower birefringence property of the sample. Further, the reconstructed 2D images of various polarization parameters such as DOP, DOLP, DOCP and anisotropy (r) from measured Stokes parameters are shown in Fig. 3.

The DOP, DOLP, DOCP and anisotropy (r) values were measured for four normal and tumour regions of ductal carcinoma tissue samples. The graphical representation of the measured polarization parameters is shown in Supplementary Figure S2. When the normal regions of ductal carcinoma tissue samples were illuminated with 0^0^ linearly polarized light, the sample exhibits greater DOP and DOLP values but barely exhibit DOCP. However, the sample exhibits comparatively lower DOLP and DOP values when incident with 90^0^ polarized light than the 0^0^ incidence. In contrast, the tumour region was found to exhibit greater DOP and DOLP values with the incidence of 90^0^ polarization light in comparison with 0^0^ polarization. Further, the normal regions of the breast tissue were found to exhibit slightly higher anisotropy than the tumour regions, corresponding to the uniform alignment of collagen in the healthy region. The reduced value of anisotropy in the tumour region is associated with the loss of collagen and also the random alignment of collagen fibers.

### Machine learning classification of polarization parameters

The feature extraction from the sample plays an important role in ML based classification and has a greater impact on the accuracy of the model. In the study, four normal and four tumour regions from ductal carcinoma tissue were selected and imaged with different input polarization states. From each of the selected images, four polarization parameters such as anisotropy, DOLP, DOCP and DOP are reconstructed. Each parameter is considered separately while training the classification model. After performing the data augmentation, the dataset contained a total of 384 image patches per parameter. Before the ML classification, 20 GLCM features are extracted from these polarization parameters. Supplementary Figures S3 and S4 represent the scatterplot for GLCM features of different polarization parameters for 0˚ and 90˚ input polarization angles.

**Fig. 4:**
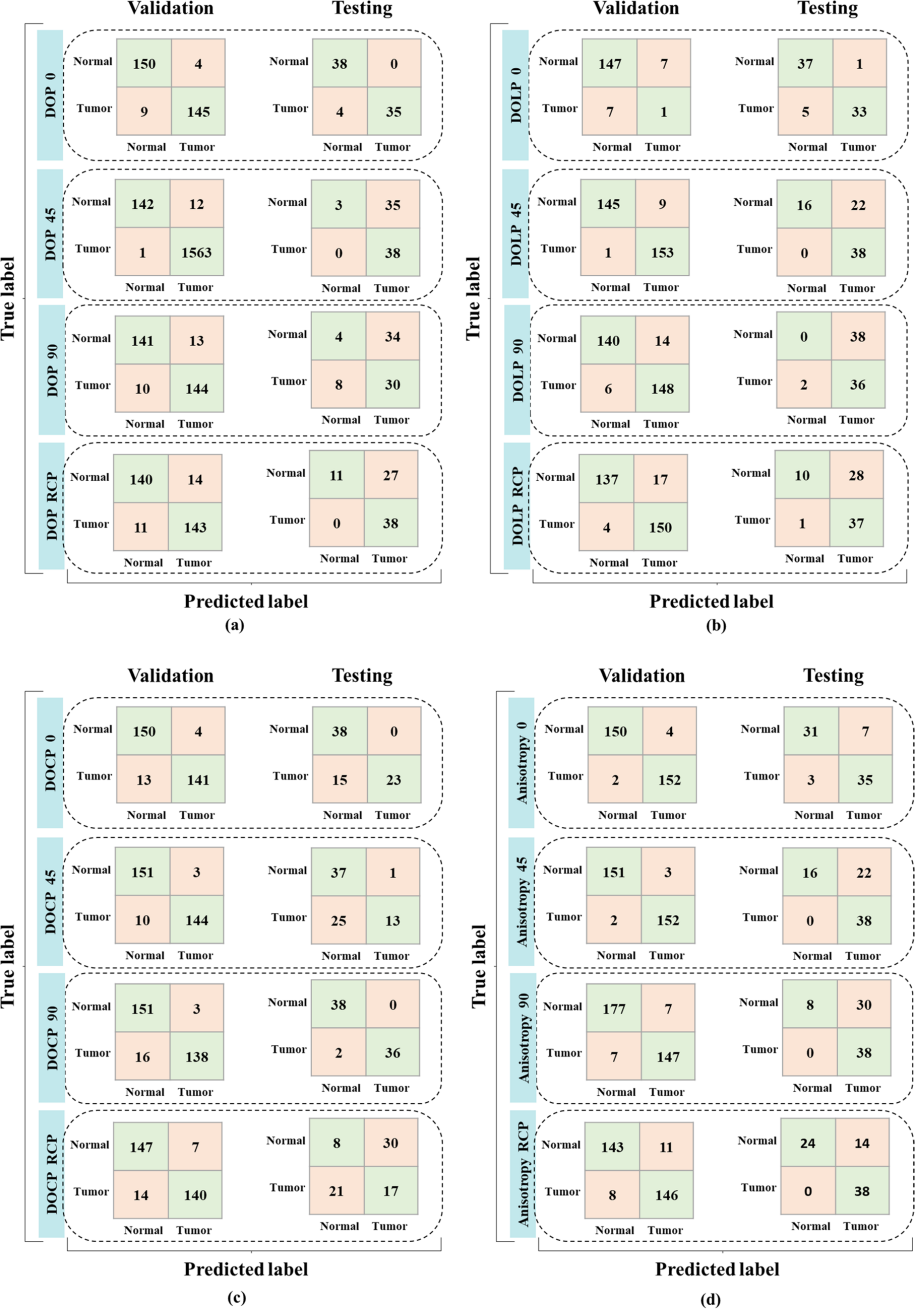
Shows the validation and testing confusion matrices for SVM classifiers trained following polarization parameter datasets with (**a**) DOP, (**b**) DOLP, (**c**) DOCP and (**d**) anisotropy

Normal/healthy regions of ductal carcinoma were found to exhibit greater DOP and DOLP values when compared to DOCP values. Also, healthy regions exhibited higher anisotropy value when compared to tumour regions of ductal carcinoma tissue. The variation in the anisotropy value corresponds to the collagen alignment of tissue structure. The normal/healthy tissue sample exhibit uniform alignment whereas malignant tissue regions suffer from loss of collagen and hence exhibit random alignment which hence reduces the birefringence nature of the sample, reducing anisotropy value. Further, the GLCM analysis also showed a similar pattern supporting the results. GLCM analysis was performed for a set of images acquired from normal and tumour regions of ductal carcinoma samples. These 20 GLCM features extracted from images of four polarization parameters namely, DOP, DOLP, DOCP and anisotropy when illuminated with 0^0^, 45^0^, 90^0^ and RCP input polarization light are used in training respective SVM classification models. All the classifiers are built with the same hyperparameters to maintain uniformity. 308 images of the dataset are used for training and the remaining 77 images are used for testing. The results of SVM training are presented in Fig. 4. The confusion matrix is used to calculate the performance matrices for each of the models and is represented in Table 1.

**Table 1:** Performance matrices for the SVM models trained with various dataset

Polarization parameter	Angle of polarization (degree)	Dataset	Sensitivity	Precision	Specificity	Accuracy	F1 score
Anisotropy	0	Validation	98.70	97.44	97.40	98.05	98.06
		Test	92.11	83.33	81.58	86.84	87.50
	45	Validation	98.70	98.06	98.05	98.38	98.38
		Test	100.00	63.33	42.11	71.05	77.55
	90	Validation	95.45	95.45	96.20	95.86	95.45
		Test	100.00	55.88	21.05	60.53	71.70
	RCP	Validation	94.81	92.99	92.86	93.83	93.89
		Test	100.00	73.08	63.16	81.58	84.44
DOP	0	Validation	94.16	97.32	97.40	95.78	95.71
		Test	89.74	100.00	100.00	94.81	94.59
	45	Validation	99.94	99.24	92.21	99.24	99.59
		Test	100.00	52.05	7.89	53.95	68.47
	90	Validation	93.51	91.72	91.56	92.53	92.60
		Test	78.95	46.88	10.53	44.74	58.82
	RCP	Validation	92.86	91.08	90.91	91.88	91.96
		Test	100.00	58.46	28.95	64.47	73.79
DOLP	0	Validation	12.50	12.50	95.45	91.36	12.50
		Test	86.84	97.06	97.37	92.11	91.67
	45	Validation	99.35	94.44	94.16	96.75	96.84
		Test	100.00	63.33	42.11	71.05	77.55
	90	Validation	96.10	91.36	90.91	93.51	93.67
		Test	94.74	48.65	0.00	47.37	64.29
	RCP	Validation	97.40	89.82	88.96	93.18	93.46
		Test	97.37	56.92	26.32	61.84	71.84
DOCP	0	Validation	91.56	97.24	97.40	94.48	94.31
		Test	60.53	100.00	100.00	80.26	75.41
	45	Validation	93.51	97.96	98.05	95.78	95.68
		Test	34.21	92.86	97.37	65.79	50.00
	90	Validation	89.61	97.87	98.05	93.83	93.56
		Test	94.74	100.00	100.00	97.37	97.30
	RCP	Validation	90.91	95.24	95.45	93.18	93.02
		Test	44.74	36.17	21.05	32.89	40.00

In the current study, the model trained with DOP images at 0^0^ input polarisation has the highest classification accuracy, implying that linearly polarised light can distinguish between normal and tumour tissue. The overall classification accuracy of the model is presented in Supplementary Figure S5. DOLP 0 and DOCP 90, in addition to the model mentioned above, demonstrated good classification accuracy; however, the latter is more likely to be over-trained with the current dataset.

### Mueller matrix analysis

The alteration in polarization property after the interaction of light with an optical system was described using the Mueller matrix. The Mueller matrix (M) was measured using the relation [42],

$$M=\left[\begin{array}{cccc}M(\mathrm{1,1})& M(\mathrm{1,2})& M(\mathrm{1,3})& M(\mathrm{1,4})\\ M(\mathrm{2,1})& M(\mathrm{2,2})& M(\mathrm{2,3})& M(\mathrm{2,4})\\ M(\mathrm{3,1})& M(\mathrm{3,2})& M(\mathrm{3,3})& M(\mathrm{3,4})\\ M(\mathrm{4,1})& M(\mathrm{4,2})& M(\mathrm{4,3})& M(\mathrm{4,4})\end{array}\right] =\left[\begin{array}{cccc}0.5\times \left({I}_{H}+{I}_{V}\right)& 0.5\times \left({I}_{H}-{I}_{V}\right)& \left[{I}_{P}-M\left(\mathrm{1,1}\right)\right]& \left[{I}_{R}-M\left(\mathrm{1,1}\right)\right]\\ 0.5\times \left({Q}_{H}+{Q}_{V}\right)& 0.5\times \left({Q}_{H}-{Q}_{V}\right)& \left[{Q}_{P}-M\left(\mathrm{2,1}\right)\right]& \left[{Q}_{R}-M\left(\mathrm{2,1}\right)\right]\\ 0.5\times \left({U}_{H}+{U}_{V}\right)& 0.5\times \left({U}_{H}-{U}_{V}\right)& \left[{U}_{P}-M\left(\mathrm{3,1}\right)\right]& \left[{U}_{R}-M\left(\mathrm{3,1}\right)\right]\\ 0.5\times \left({V}_{H}+{V}_{V}\right)& 0.5\times \left({V}_{H}-{V}_{V}\right)& \left[{V}_{P}-M\left(\mathrm{4,1}\right)\right]& \left[{V}_{R}-M\left(\mathrm{4,1}\right)\right]\end{array}\right]$$

**Fig. 5:**
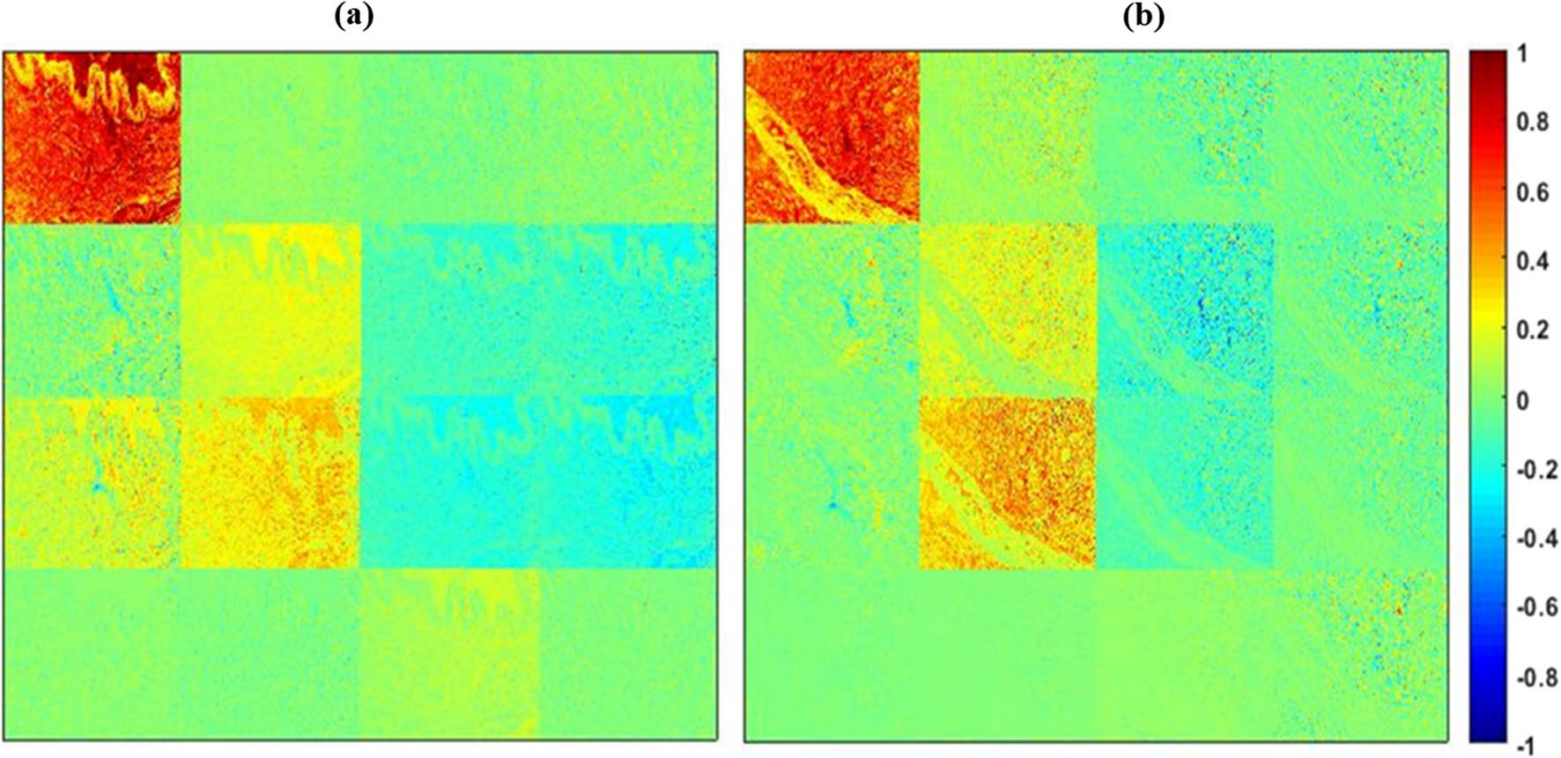
Shows 2D reconstructed Mueller matrix images for (**a**) normal region and (**b**) tumour regions of ductal carcinoma sample. The colour bar shows the value ranging from -1 to 1

Here, the subscripts H, P, V, and R correspond to the measured Stokes parameters for the polarization states, 0^0^, 45^0^, 90^0^ and RCP of incident light. All the components of the Mueller matrices were normalized with respect to the element M(1,1). The normal and tumour regions of the ductal carcinoma tissue sample were illuminated with light of various polarization states and the corresponding output polarization state was measured. The Mueller images were reconstructed using MATLAB software as shown in Fig. 5.

From Fig. 5, it is evident that M(2,4) and M(3,4) of the normal region exhibits lower value when compared to the tumour region, whereas, M(3,1) from the normal region exhibits slightly higher value than the tumour region. However, the occurrence of several scattering effects in a complex medium such as biological tissues leads to complexities in the measurement. In this regard, assessment of biological tissue through polar decomposition of the Mueller matrix approach, where three basis matrices can be used as an effective tool to distinguish multiple scattered light and to gain individual polarization properties. Polarization properties such as retardance, diattenuation and depolarization can be used for investigating the composition and microstructure of biological tissue which could be beneficial in image-guided therapy and tissue diagnosis. For a normal region of ductal carcinoma sample the Mueller matrix (M), diattenuation matrix ${(M}_{D}),$ depolarization matrix (M_∆_) and retardance matrix ${(M}_{{\text{R}}})$ obtained are as follows,
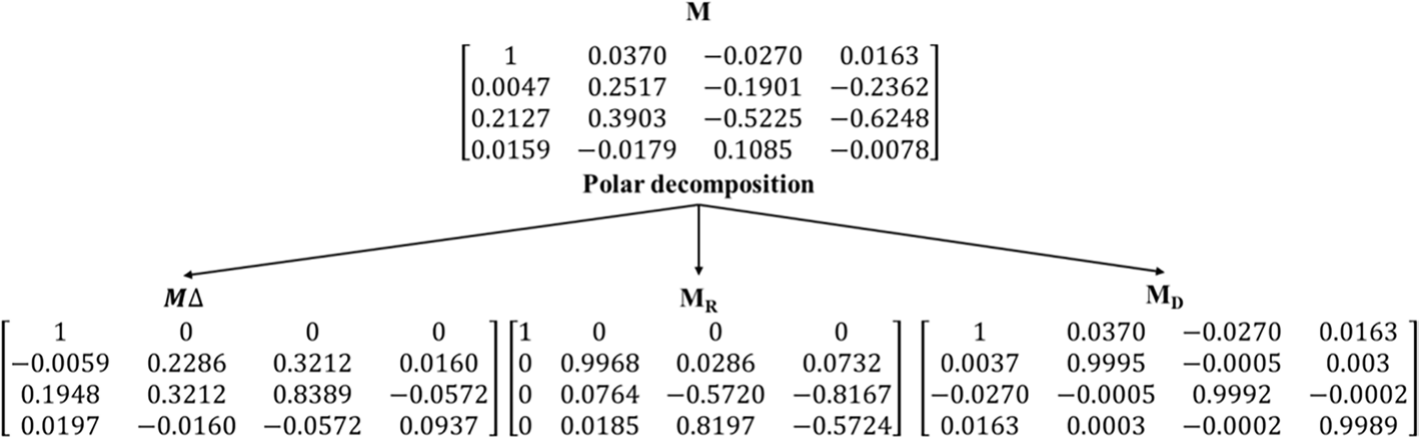


Similarly, for tumour regions of ductal carcinoma sample the Mueller matrix (M), diattenuation matrix ${(M}_{D}),$ depolarization matrix (M_∆_) and retardance matrix ${(M}_{{\text{R}}})$ obtained are as follows,
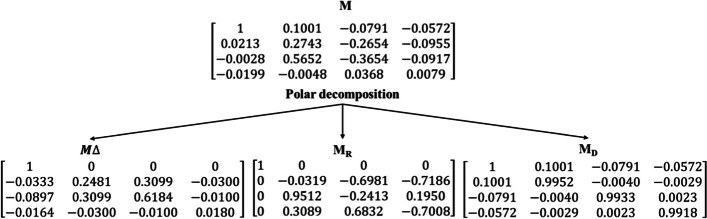


Depolarization occurs due to multiple scattering phenomena of photons arising from a difference in refractive indices of the various structures present in the sample, which affects the output polarization state. During scattering, the polarization states of photons become increasingly random relative to one another. Depolarization of tissue is greatly dependent on the composition or morphology of tissue samples and studies have shown the correlation of depolarization with cancer grading. The normal regions of ductal carcinoma tissue specimens were found to exhibit an average depolarization value, ∆ = 0.644 and tumour regions exhibited a slightly increased depolarization value of 0.826. Linear retardance (birefringence) is another important polarimetric effect within biological tissues. The normal/healthy tissue sample exhibit uniform alignment, whereas abnormal tissue regions suffer from loss of collagen and random alignment affecting the birefringence nature of the sample, hence reducing retardance values. In our study, the normal region is found to have a linear retardance (δ) value of 2.373 whereas tumour regions showed reduced retardance value of 0.748. The reduction in retardance value is associated with the alignment of fibrillar collagen present in the tissue sample. In biological tissues, there is often a negligible effect due to diattenuation than depolarization or retardance. However, normal regions were found to have lower diattenuation (d = 0.043) value when compared to tumour regions (d = 0.11). Supplementary Figure S6 shows the graphical representation of decomposition parameters for both normal and tumour regions.

At present, Stokes-Mueller imaging is acquiring appreciable attention as it is favourable for biomedical applications for various reasons: (i) it characterizes the morphological changes in the tissue structure by exploiting the polarization property of light; (ii) being a non-invasive technique, it acquires tissue images without any external labelling; (iii) it can be designed by using inexpensive light sources such as LEDs which are harmless for samples and to patients at optimal energy; (iv) simultaneous measurement of Stokes vector and Mueller matrix will reduce experimental time to a great extent. Stokes-Mueller polarimetric methods have several advantages over other imaging modalities such as being more sensitive to larger sampling depths, less complex and less expensive. Manual image analysis of tissue samples under a microscope is very tedious and time-consuming due to the complex nature of biological entities, which in turn demands an expert pathologist to achieve accurate output. To overcome these limitations, an automatic fast and robust image processing technique is desirable. ML algorithms-based classification reveals the important features of the sample under study, and also performs well with smaller datasets.

### Machine learning analysis of Mueller matrix

For the study, all Mueller matrix component is treated as distinct entity. Each image is divided into 16 equal segments as a part of data augmentation, and the GLCM parameters are computed from the individual image patches. This is similar to how the polarization parameter analysis is performed. Consequently, we obtained 6144 image patches in total, of which 4916 are used for training and validation, and the remaining ones are used for model testing. The trained SVM model displayed 83% validation accuracy and 53% testing accuracy as represented in Supplementary Figure S7.

## Conclusion

The Stokes-Mueller based imaging system in transmission geometry could become a promising tissue imaging technique, due to its potential to measure polarization dependence on the structural alignment of tissue. Even a slight variation in structural alignment induces a change in polarization property, which hence plays a crucial role in the early detection of abnormal tissue morphology. In the present study, tissue images from both normal and tumour regions of ductal carcinoma tissue are captured using the Stokes-Mueller polarization microscope with various input polarization states and corresponding polarization parameters were measured. The polarization signal depends on the orientation of molecules; hence Mueller imaging exhibits significant enhancement of contrast from fibrous structures which is hardly seen under conventional polarization imaging. The robust technique enables the automatic analysis of images with minimum human intervention to increase classification accuracy. However, development is needed to improve model performance by finding significant features for classification. Further, the ML-based image classification model is not fully automated and requires manual feature extraction. With the acquired knowledge regarding the sample features, it is easier to extend the classification problems from machine learning to deep learning which is a widely used image analysis technique. This step gives a deeper vision regarding significant features of the sample under study. Further, the Stokes-Mueller polarization imaging system in reflection geometry can be used to study the tissue property in vivo. The proposed measurements of Stokes vectors and Mueller matrices may enable us to investigate the interaction of polarized lights with biomolecules in biopsy tissue specimens and could find its usefulness in the diagnosis of clinical conditions such as Alzheimer, Diabetes, Cancer, and wound healing.

## Supplementary Information

Below is the link to the electronic supplementary material.

Supplementary file1 (DOCX 3590 KB)
