# Supplementary material for: Characterization and classification of ductal carcinoma tissue using four channel based stokes-mueller polarimetry and machine learning
Source: Lasers Med Sci. 2024 May 4;39(1):123. doi: 10.1007/s10103-024-04056-5 (PMC11069477; doi:10.1007/s10103-024-04056-5)
Supplement: Supplementary file 1 — Supplementary file1 (DOCX 3590 KB) [file 10103_2024_4056_MOESM1_ESM.docx]

**Supplementary data**


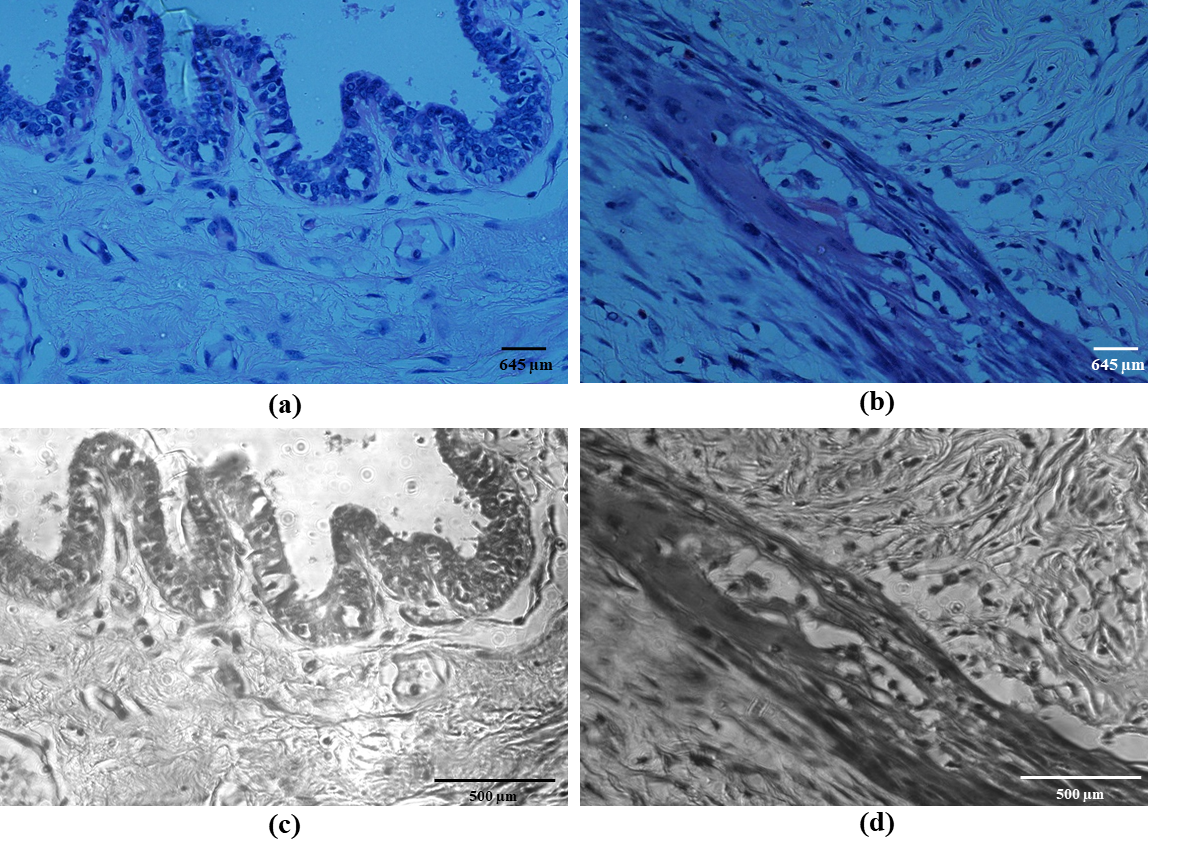


**Figure S1.** (a, b) Brightfield image captured using Olympus BX51, (c, d) polarization image captured using developed polarization microscope from normal and tumour regions of ductal carcinoma tissue, respectively.

**
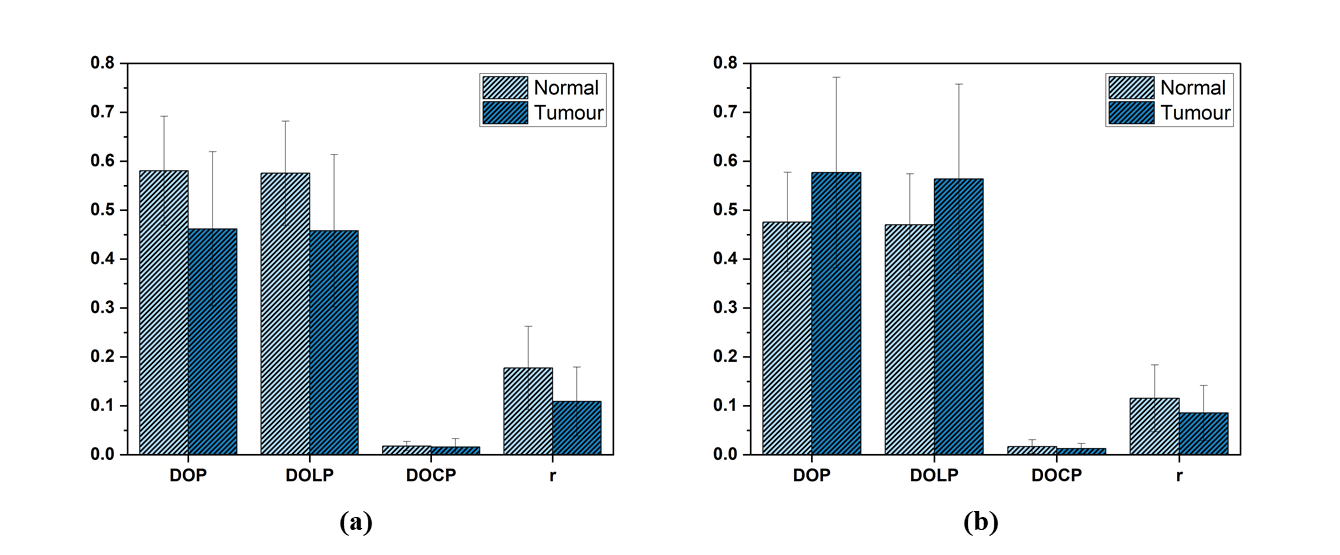
**

**Figure S2**. Shows the graphical representation of polarization parameter values for normal and tumour regions illuminated with (a) 0⁰ and (b) 90⁰ polarization.

**
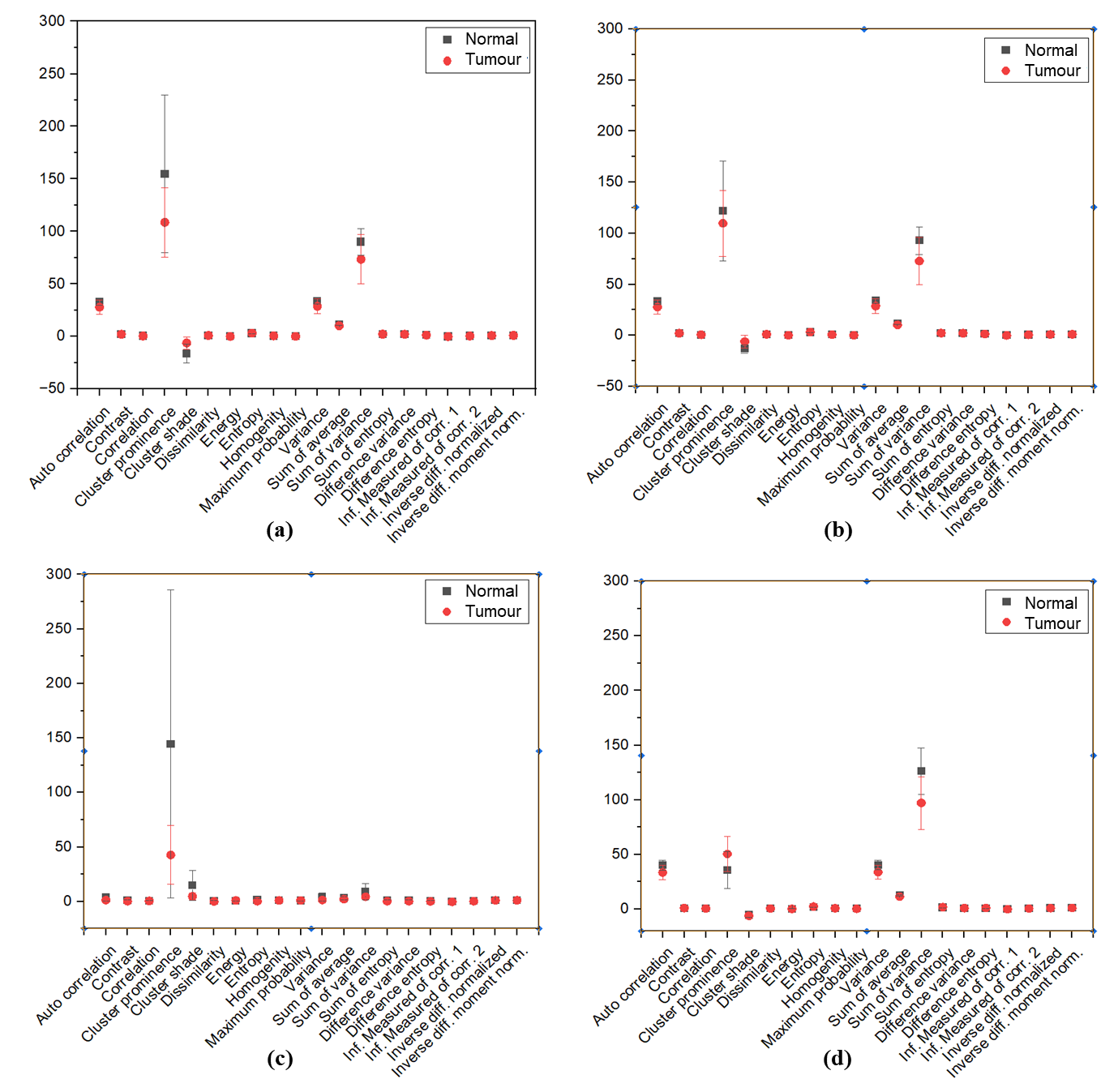
**

**Figure S3.** Shows the scatter plots representing GLCM features of the polarization parameters (a) DOP, (b) DOLP, (c) DOCP and (d) anisotropy measured at 0⁰ linearly polarized light.

**
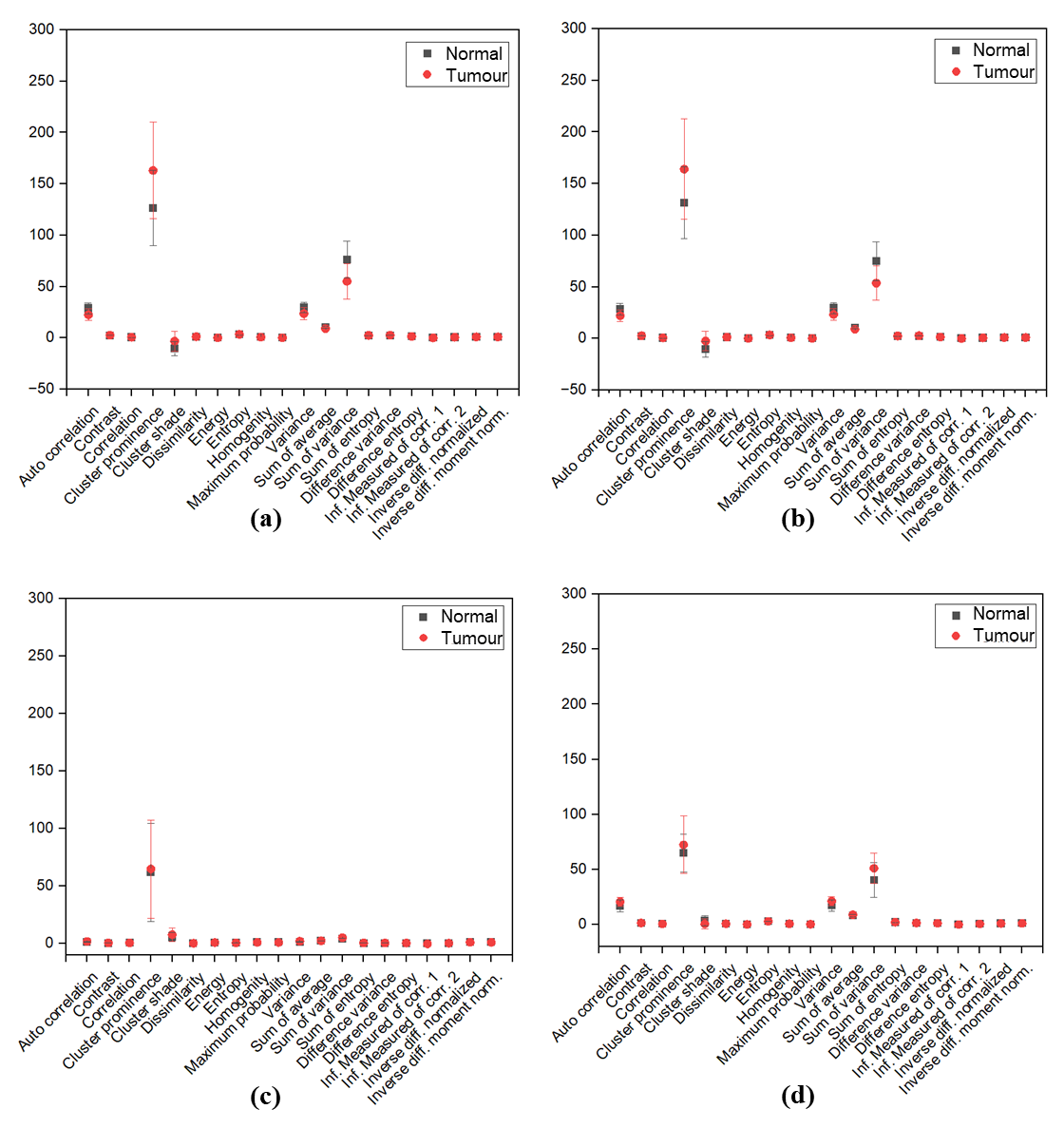
**

**Figure S4.** Shows the scatter plots representing GLCM features of the polarization parameters (a) DOP, (b) DOLP, (c) DOCP and (d) anisotropy measured at 90⁰ linearly polarized light.

**
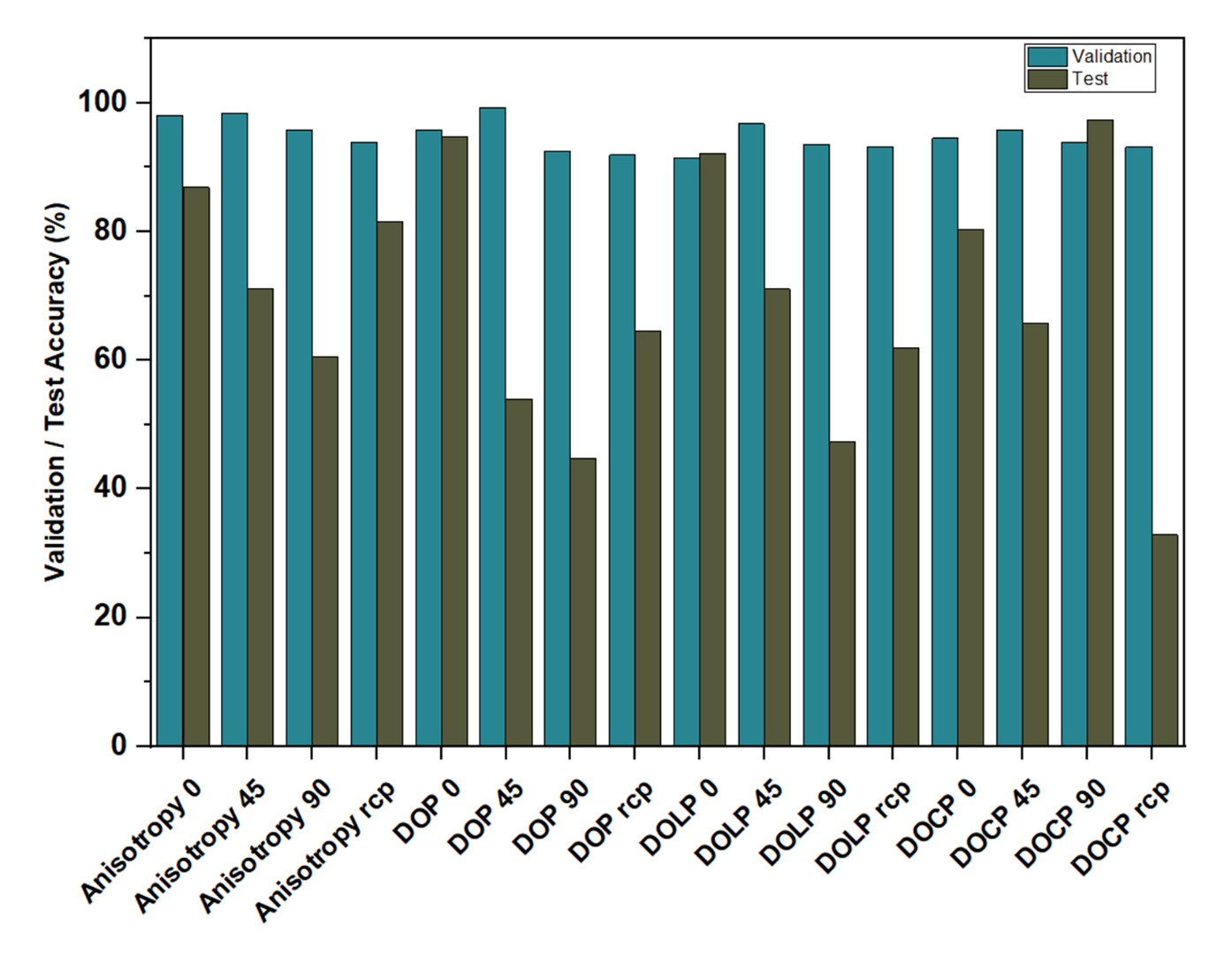
**

**Figure S5.** Shows the validation and testing classification accuracy for various SVM models.


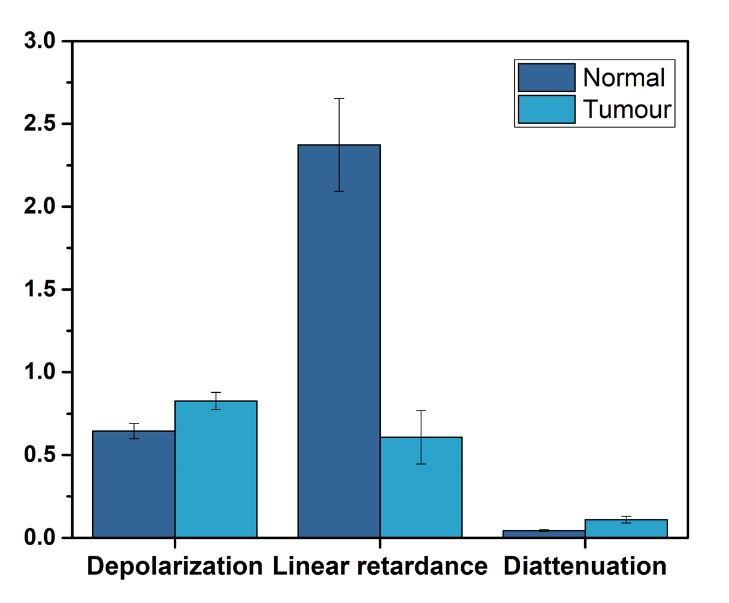


**Figure S6.** Shows graphical representation of Lu-Chipman decomposition parameter values for normal and tumour region.


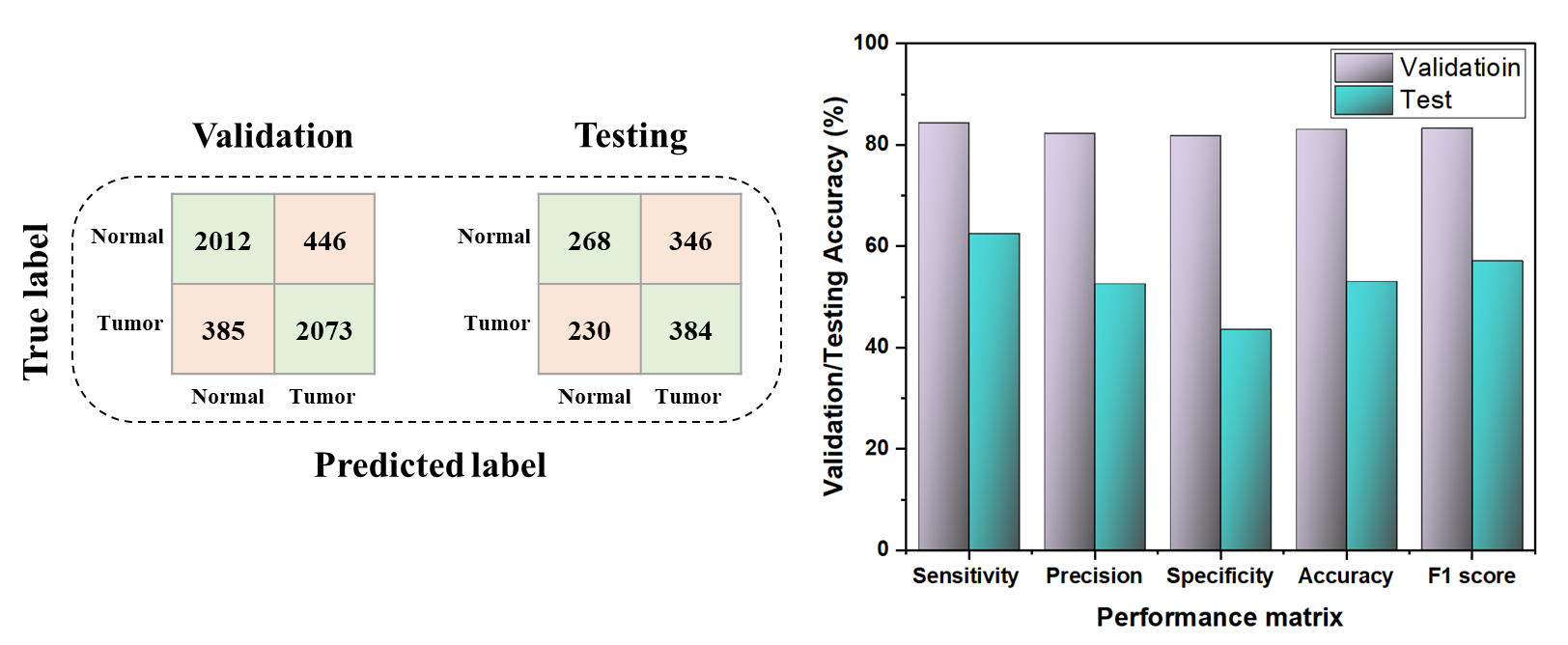


**Figure S7.** Shows confusion matrix and performance matrices for model trained with Mueller images.
